# Supplementary material for: Human Amniotic Membrane Mesenchymal Stem Cell-Synthesized PGE2 Exerts an Immunomodulatory Effect on Neutrophil Extracellular Trap in a PAD-4-Dependent Pathway through EP2 and EP4
Source: Cells. 2022 Sep 10;11(18):2831. doi: 10.3390/cells11182831 (PMC9496826; doi:10.3390/cells11182831)
Supplement: Supplementary file 1 [file cells-11-02831-s001.zip › cells-1832656-supplementary.pdf]

Supplementary Figures

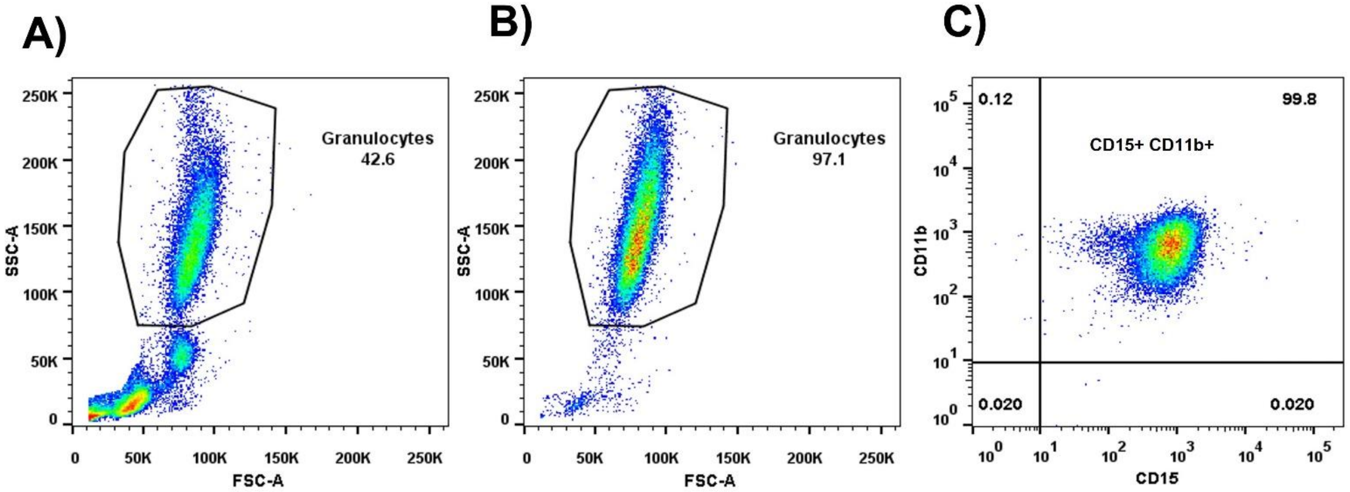

Figure S1. Human neutrophils isolation purification corroboration. Peripheral blood from healthy volunteers were obtained from the cubital vein in the presence of anticoagulant. More than 40% of cellular elements were neutrophils according to their SSC-A and FSC-A features (A). After polymorphoprep purification, there were up to 97% of granulocytes according to their SSA-A and FSC-A features (B); from those granulocytes (shown in B) up to 99% were $CD11^{+}CD15^{+}$ neutrophils. Representative flow cytometry dot plots from 3 independent assays.

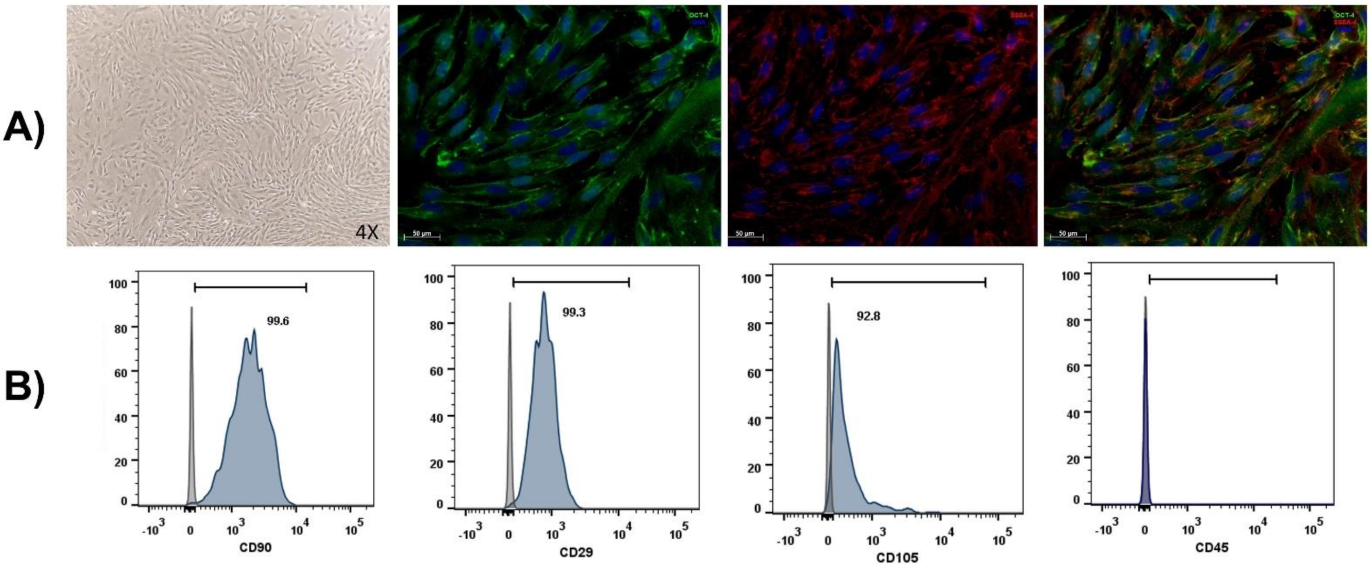

Figure S2. Human amniotic membrane cells characterization. Human amniotic membrane was obtained from the amniotic membrane tissue bank of the Institute of Ophthalmology Conde de Valenciana Foundation. Cells attached to the plastic and presented a fibroblast like morphology (left image, panel A. Inverted microscopy image). Cells expressed the embryonic SSEA-4 and Oct-4 transcription factors (second and third images, from left to right, panel A). Merged image representing the concomitant expression of both SSEA-4 and Oct-4 stem cells markers (fourth image from left to right, panel A). Representative

images from 3 independent assays. Scale bars represent 50 μm. Cells were obtained from the cultures and stained with fluorochrome-conjugated antibodies. All the cells were > 90% positive to CD90, CD29 and CD105 markers and negative to the pan-leucocyte marher CD45 (B). Left gray peaks represent negative controls. Representative histograms from 3 independent assays.

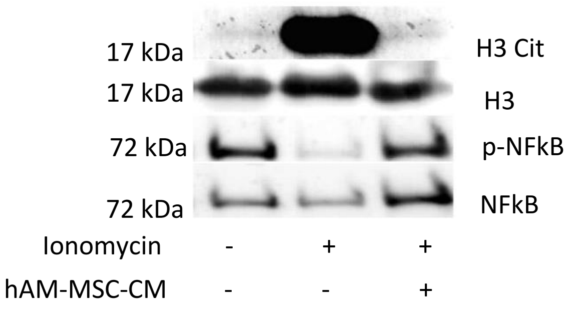

Figure S3. hAM-MSC-CM is able to reduce PAD-4 activity and prevent NFkB activation reduction. Protein extracts from neutrophils incubated in the mentioned conditions were probed with the aforementioned antibodies. As expected, ionomycin was able to induce H3 citrullination and interestingly, the concomitant incubation of neutrophils with ionomycin and hAM-MSC-CM considerably reduced citrullination comparable to basal (unstimulated) conditions. Also, ionomycin was able to reduce NFκB phosphorylation, which was prevented with concomitant incubation with ionomycin and hAM-MSC-CM.
